# Type VII Secretion Substrates of Pathogenic Mycobacteria Are Processed by a Surface Protease

**DOI:** 10.1128/mBio.01951-19

**Published:** 2019-10-29

**Authors:** Maroeska J. Burggraaf, Alexander Speer, Aniek S. Meijers, Roy Ummels, Astrid M. van der Sar, Konstantin V. Korotkov, Wilbert Bitter, Coenraad Kuijl

**Affiliations:** a Amsterdam UMC, Vrije Universiteit Amsterdam, Medical Microbiology and Infection Control, Cancer Center Amsterdam, Amsterdam, Netherlands; b Amsterdam UMC, Vrije Universiteit Amsterdam, Medical Microbiology and Infection Control, Amsterdam Institute of Infection & Immunity, Amsterdam, Netherlands; c Molecular and Cellular Biochemistry, College of Medicine, University of Kentucky, Lexington, Kentucky, USA; d Molecular Microbiology, Vrije Universiteit Amsterdam, Amsterdam, Netherlands; Washington University School of Medicine in St. Louis

**Keywords:** aspartic protease, *Mycobacterium*, PE proteins, PE_PGRS, type VII secretion

## Abstract

Tuberculosis, one of the world’s most severe infectious diseases, is caused by Mycobacterium tuberculosis. A major weapon of this pathogen is a unique cell wall that protects the pathogen from eradication by the immune system. Mycobacteria have specialized secretion systems, e.g., type VII secretion or ESX systems, to transport substrates across this cell wall. The largest group of proteins that are secreted by these ESX systems are the PE proteins. Previously, it was shown that the N-terminal PE domain of about 100 amino acids is required for secretion. Here, we describe the identification of an aspartic protease, designated PecA, that removes (part of) this PE domain at the cell surface. Nearly all of the observed PE_PGRS proteins are processed by PecA. Interestingly, the protease itself is also a secreted PE protein and subject to self-cleavage. Furthermore, a defect in surface processing has no effect on the activity of the PE lipase protein LipY but does seem to affect the functioning of other virulence factors, as a *pecA* mutant strain of Mycobacterium marinum shows moderate attenuation in zebrafish larvae. In conclusion, our results reveal the presence of a functional aspartic acid protease in M. marinum that cleaves LipY, itself as well as other members of the PE_PGRS family. Finally, mutants lacking PecA show growth attenuation *in vivo*, suggesting that PecA plays a role during infection.

## Abstract (precis)

Aspartic proteases are common in eukaryotes and retroviruses but are relatively rare among bacteria (N. D. Rawlings and A. Bateman, BMC Genomics 10:437, 2009, https://doi.org/10.1186/1471-2164-10-437). In contrast to eukaryotic aspartic proteases, bacterial aspartic proteases are generally located in the cytoplasm. We have identified a surface-associated mycobacterial aspartic protease, PecA, which cleaves itself and many other type VII secretion substrates of the PE_PGRS family. PecA is present in most pathogenic mycobacterial species, including M. tuberculosis. In addition, pathogenicity of M. marinum is reduced in the *ΔpecA* mutant, indicating that PecA contributes to virulence.

## INTRODUCTION

Tuberculosis is one of the world’s deadliest infectious diseases with 1.3 million deaths each year (1). Its causative agent is Mycobacterium tuberculosis, a facultative intracellular pathogen with a highly unique diderm cell envelope. This unique cell envelope is highly impermeable and requires specialized secretion systems for protein transport to the cell surface (2, 3). These protein secretion systems are generally known as type VII secretion systems, and pathogenic mycobacteria have up to 5 of these systems, e.g., ESX-1 to ESX-5 (4). ESX-1 and ESX-5 are the best-studied systems. ESX-1 is known for its role in virulence, since the deletion of part of the *esx-1* locus, known as the RD-1 region, is the main cause of the attenuation of the Mycobacterium bovis BCG vaccine strain (5, 6). ESX-5 is required for the secretion of more than 50 proteins (7, 8) and has been described to be important for immune modulation (9–11) and nutrient uptake (12). Notably, the ESX-5 system is present only in slow-growing mycobacteria (13).

Together with the diversification of ESX systems, two large groups of proteins have evolved, known as the PE and PPE proteins. PE/PPE proteins are unique to mycobacteria and some closely related species and are especially abundant in pathogenic slow-growing species like M. tuberculosis and Mycobacterium marinum (14). Some of the PE- and PPE-encoding genes are present in the *esx* loci, while others are located elsewhere in the genome (15). These proteins are prominent ESX substrates, and the majority of these proteins are secreted through the ESX-5 system (7, 16). The currently available structural data show that PE and PPE proteins form heterodimers (17–19), which is hypothesized to be a general feature of these proteins.

PE/PPE proteins are characterized by a conserved N-terminal domain of 100 and 180 amino acids (aa), respectively, which includes the Pro-Glu or Pro-Pro-Glu motifs close to the N terminus after which they were named (20). Although both these protein families lack a classical signal peptide, the PE proteins do have a conserved ESX secretion motif, consisting of a helix-turn-helix domain directly followed by YxxxD/E (17).

Some PE proteins contain only a PE domain (PE_only_), while others have C-terminal extensions of variable size. The largest subfamily of PE proteins in M. tuberculosis contains a glycine-rich C-terminal domain encoded by so-called polymorphic GC-rich repetitive sequences (PGRS) and are therefore known as PE_PGRS proteins. These proteins have been linked to immune modulation (9–11). Although the high prevalence of PE_PGRS proteins in pathogenic mycobacteria and their secretion by the ESX-5 system hint at a role in virulence, the regulation and processing of this group of proteins remain unclear.

**FIG 1:**
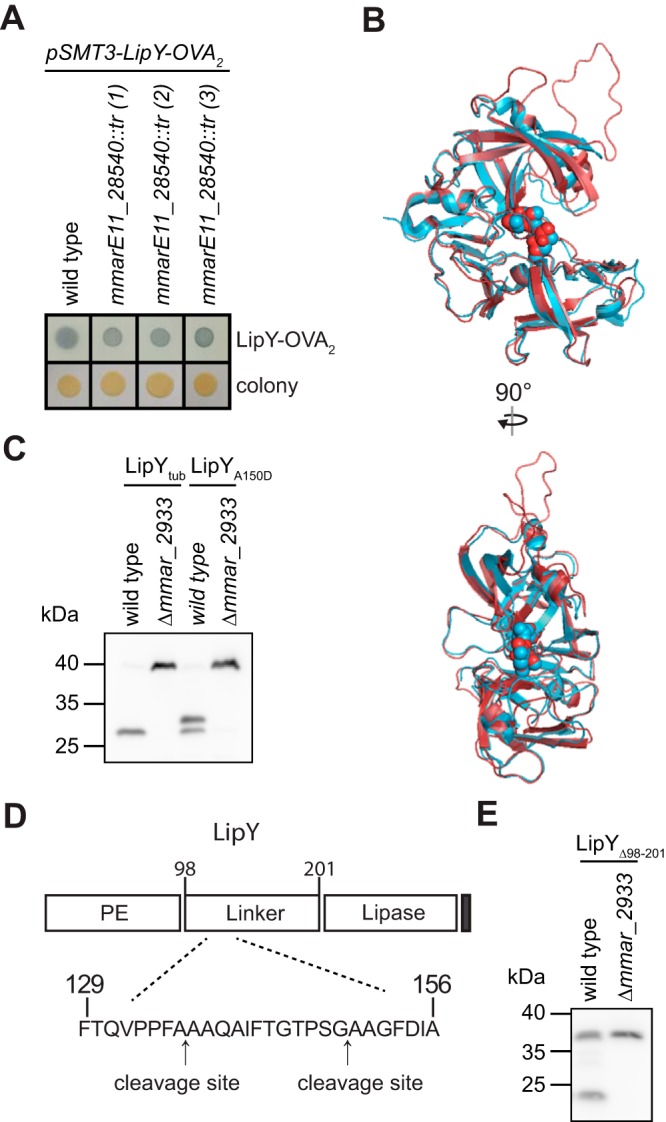
MMARE11_28540 is required for the processing of LipY. (A) Secretion of LipY-OVA_2_ by M. marinum wild-type E11, *mmarE11_28540*::*tr*, and complemented strains was detected by double filter assay. (B) Alignment between model of MMARE11_28540 (red, amino acids 269 to 551 out of 554) and crystal structure of PE_PGRS16 (blue, 4EHC). Aspartic acids within the putative active site DTG/DSG motifs are depicted as spheres. (C) Immunoblot analysis of whole-cell lysate from M. marinum M wild-type and *Δmmar_2933* strains expressing C-terminally HA-labeled LipY_tub_ or LipY_A150D_. LipY was detected with anti-HA antibody. (D) The cleavage sites of MMAR_2933 in the linker domain of LipY_tub_ as determined by N-terminal Edman sequencing; the N-terminal cleavage site (aa 136A to 137A) and the previously identified C-terminal cleavage (aa 149G to 150A) (23) in LipY_tub_ are indicated. (E) Immunoblot analysis of whole-cell lysate from M. marinum M wild-type and *Δmmar_2933* strains expressing C-terminally HA-labeled LipY_Δ98-201_. Proteins were detected with anti-HA antibody.

One of the characterized secreted PE proteins is the lipase LipY from M. tuberculosis (LipY_tub_). LipY_tub_ functions as a surface-exposed triacylglycerol lipase. In M. tuberculosis, LipY is mainly expressed under hypoxic conditions and could be involved in lipid body formation (21, 22). LipY consists of a PE domain followed by a linker domain and the lipase domain (Fig. 1D). Previously, we showed that LipY_tub_ is secreted by ESX-5 and subsequently processed in the linker domain (23).

In this study, we describe the identification and characterization of an aspartic protease, which we named PecA, that processes LipY_tub_ and other PE_PGRS proteins. Interestingly, deletion of *pecA* causes attenuation of M. marinum in zebrafish larvae.

## RESULTS

### Identification of MMARE11_28540.

To study the secretion and processing of PE proteins, we used M. marinum and heterologous expression of the model protein LipY-OVA_2_, consisting of the PE domain of LipY linked to an ovalbumin fragment. Previously, we have shown that this fusion construct is secreted, which can be detected by double filter assay (24). To identify proteins involved in PE protein secretion, we generated and screened a transposon mutant library of M. marinum expressing LipY-OVA_2_. We expected to find mutants that were blocked in secretion because they contained a mutation in the gene coding for the LipY partner protein, but we did not identify these. Instead, we identified three transposon mutants that reproducibly displayed more intense and more discrete edges on the double filter than the wild-type strain (Fig. 1A).

Analysis of the transposon insertions revealed that all 3 mutants had independent transposon insertions in the same gene, i.e., *MMARE11_28540.* The encoded protein belongs to the PE_PGRS family and is 69.6% identical to PE_PGRS35 (Rv1983) of M. tuberculosis. Somewhat weaker similarity (43% identity) is observed with the C-terminal domain of PE_PGRS16. Interestingly, the structure of the C-terminal domain of PE_PGRS16 has been solved and belongs to the family of aspartic proteases (25). Although the crystal structure of PE_PGRS16 was solved, no proteolytic activity was observed and putative substrates remained elusive. Based on homology modeling, MMARE11_28540 has a similar structure, including the location of the DTG/DSG motifs that form the active site of aspartic proteases (Fig. 1B; see also Fig. S1A and B in the supplemental material).

10.1128/mBio.01951-19.1 FIG S1 Comparison of the aspartic protease domains. (A) Structural comparison of the protease domains of PE_PGRS16 and MMARE11_28540. Side and front views depicting the crystal structure of protease domains of PE_PGRS16 (PDB accession no. 4EHC, excluding His tag) and a model of MMARE11_28540 (amino acids 269 to 551 out of 554). Aspartic acids within the active site containing DTG/DSG motifs are shown in orange. (B) Sequence alignment performed by MUSCLE of putative aspartic proteases (domains) in M. tuberculosis, M. marinum, human pepsinogen A3 (PGA3), and HIV protease (HIV-PR). Boldface indicates identified protease that affects LipY_tub_ processing. Underscoring indicates that aspartic acid 293 is mutated to glycine when referring to the catalytic dead mutant D293G. Red indicates the aspartic acid signature DTG/DSG motifs. The HIV protease sequence is repeated to simulate the two lobes found in the other proteases. Download FIG S1, PDF file, 1.9 MB. Copyright © 2019 Burggraaf et al. 2019 Burggraaf et al. This content is distributed under the terms of the Creative Commons Attribution 4.0 International license.

### LipY processing.

To assess the function of MMARE11_28540, we first analyzed the effect of this gene on full-length LipY_tub_. Previously, we have shown that LipY_tub_ is cleaved in the linker domain after amino acid 149 (23). For follow-up experiments, we generated a clean deletion mutant of the *mmarE11_28540* orthologue in M. marinum M (*mmar_2933*). We expressed LipY_tub_ with a C-terminal hemagglutinin (HA) tag in M. marinum wild type and the *Δmmar_2933* strain. Western blot analysis showed that deletion of *mmar_2933* completely abolished the processing of LipY_tub_ (Fig. 1C), indicating that processing of LipY_tub_ is performed by MMAR_2933. From our previous experiments, we knew that there is an additional cleavage site in LipY_tub_, because mutation of the original cleavage site (by the A150D mutation) resulted in a slightly larger but still cleaved product of around 33 kDa (Fig. 1C) (23). Interestingly, cleavage at this additional site was also not observed in the *mmar_2933* mutant, which indicates that processing at this alternative site is also mediated by this putative protease.

To determine the localization of this additional cleavage site, we isolated processed LipY_A150D_ from the Genapol supernatant (GS) (Fig. S2) and performed Edman sequencing. The resulting sequence showed that LipY_A150D_ is cleaved between two alanine residues at positions 136 and 137, resulting in the N-terminal sequence AAQAI (Fig. 1D). This cleavage site corresponds to the observed molecular weight of the processed fragment (∼33 kDa).

10.1128/mBio.01951-19.2 FIG S2 Processing of LipY. Genapol supernatant fractions of M. marinum M wild type and wild type expressing *pSMT3-lipY_A150D_* were stained with Coomassie brilliant blue R250. The arrow indicates the protein band that was subjected to Edman sequencing. Download FIG S2, PDF file, 0.3 MB. Copyright © 2019 Burggraaf et al. 2019 Burggraaf et al. This content is distributed under the terms of the Creative Commons Attribution 4.0 International license.

Cleavage of both LipY_tub_ and LipY_A150D_ takes place in the linker domain. However, LipY-OVA_2_, whose secretion was affected in *mmarE11_28540*::*tr*, does not contain this linker domain. This hinted at the possibility that MMAR_2933 also cleaves at a third location, inside the PE domain of LipY-OVA_2_. To explore cleavage inside the PE domain, we expressed LipY without the linker domain (LipY_Δ98–201_) in wild-type and *Δmmar_2933* strains. In the wild-type strain, we observed both the full-length product of LipY_Δ98–201_ at 36 kDa and a processed fragment with an apparent weight of 25 kDa (Fig. 1E). Similarly to the full-length protein, this shorter LipY_tub_ product was not processed in the *Δmmar_2933* mutant strain. This indicates that MMAR_2933 can cleave LipY both inside the PE domain, before amino acid 98, and after amino acids 136 and 149. Strikingly, the apparent molecular weight of the N-terminal part that is cleaved off (11 kDa) corresponds to the size of the PE domain. These experiments demonstrate that MMAR_2933 is a protease that cleaves PE proteins, and therefore, we would like to rename this protein PE cleavage protein A (PecA).

### LipY surface localization and activity.

**FIG 2:**
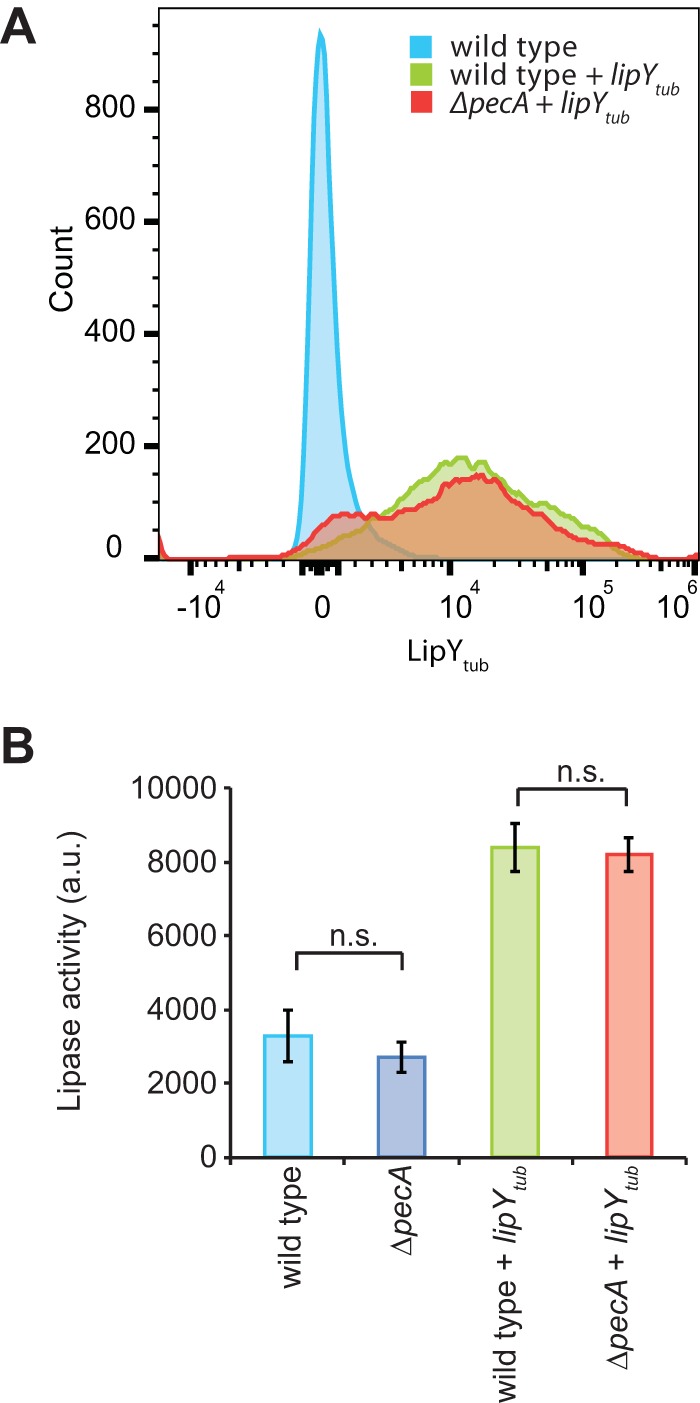
PecA does not affect LipY surface localization and lipase activity. (A) Surface localization of LipY was measured on whole cells by flow cytometry for M. marinum M wild-type and *ΔpecA* strains expressing LipY_tub_. LipY_tub_ was detected via its C-terminal HA tag by anti-HA antibody (10,000 bacteria were counted per condition). (B) Lipase activity was measured by the fluorescently based DGGR assay. Standard deviation values are shown (*n* = 3). a.u., arbitrary units; n.s., not significant.

Since the majority of LipY in the Genapol supernatant fraction is processed by PecA, we questioned whether LipY processing is required for proper surface localization. This could also elucidate at which cellular location cleavage is taking place. Surface localization of LipY_tub_ was analyzed by flow cytometry. This experiment showed that cell surface exposures of LipY_tub_ were similar for the wild-type and the *ΔpecA* mutant strains (Fig. 2A), demonstrating that cleavage is not required for secretion and is likely to occur at the cell surface.

Previous research has shown that the presence of the PE domain reduces the lipase activity of recombinant LipY_tub_ produced in Escherichia coli (26). To test whether the presence of the PE domain reduces lipase activity in its native environment, we analyzed the lipase activity of LipY_tub_ in M. marinum wild-type and *ΔpecA* strains. Surprisingly, no change in lipase activity was observed between the wild type and the *ΔpecA* strain overexpressing LipY_tub_ (Fig. 2B). Furthermore, lipase activity increased 2.7-fold upon LipY_tub_ overexpression. Lipase activity was almost fully inhibited upon treatment with the lipase inhibitor paraoxon (POX) (Fig. S3), demonstrating the specificity of the lipase assay. Overall, our evidence suggests that processing of LipY_tub_ by PecA does not affect LipY_tub_ lipase activity in M. marinum.

10.1128/mBio.01951-19.3 FIG S3 Lipase activity of M. marinum wild-type and *ΔpecA* strains with or without expression of LipY_tub_. Lipase activity was measured by a DGGR assay. No lipase activity was detected following treatment with lipase inhibitor POX. Data are presented as mean ± SD values (*n* = 3). Download FIG S3, PDF file, 0.1 MB. Copyright © 2019 Burggraaf et al. 2019 Burggraaf et al. This content is distributed under the terms of the Creative Commons Attribution 4.0 International license.

### PecA cleavage.

**FIG 3:**
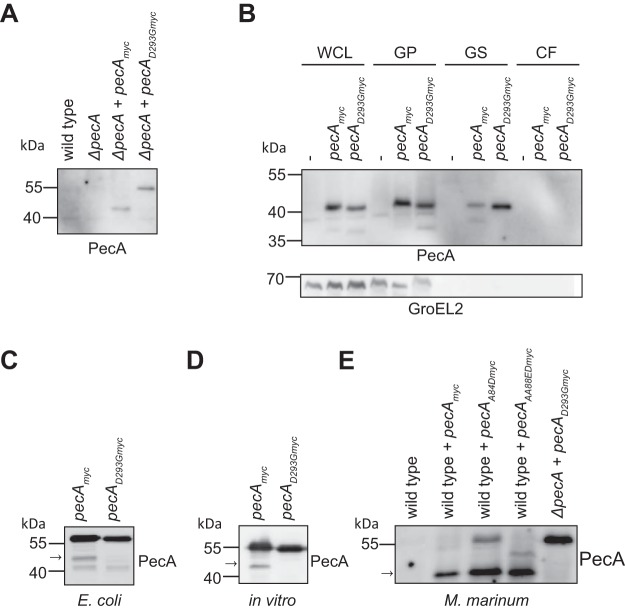
PecA is subjected to self-cleavage. (A) Expression of PecA_myc_ and PecA_D293Gmyc_ in M. marinum *ΔpecA* was detected by immunoblotting of whole-cell lysate. (B) Subcellular localization and secretion analysis of M. marinum wild type (indicated with “-”) or wild type expressing PecA_myc_ or PecA_D293Gmyc_. WCL, whole-cell lysate; GP, Genapol-treated pellet (membrane associated and intracellular); GS, Genapol supernatant (membrane associated); CF, culture filtrate. GroEL2 was probed as a cytosolic control. (C) Immunoblot analysis of PecA_myc_ and PecA_D293Gmyc_ expressed in E. coli (whole-cell lysate). (D) Immunoblot analysis of *in vitro* translation of PecA_myc_ and PecA_D293Gmyc_. (E) Immunoblot analysis of M. marinum wild type; wild type expressing PecA_myc_, PecA_A84Dmyc_, or PecA_AA88EDmyc_; and *ΔpecA* mutant expressing PecA_D293Gmyc_ (whole-cell lysate). The arrow indicates the fully cleaved form of PecA. Proteins were detected by their C-terminal Myc tag with an anti-Myc antibody.

PecA is a PE_PGRS protein and has a similar domain structure as LipY_tub_: a PE domain followed by a linker domain and a C-terminal domain with enzymatic activity. To investigate whether PecA has the capacity to cleave itself, PecA with a C-terminal Myc tag was introduced in the M. marinum *ΔpecA* strain. Western blot analysis showed that PecA had an observed molecular weight of approximately 42 kDa, instead of the expected 53 kDa, suggesting the removal of the 11-kDa PE domain (Fig. 3A).

The active site of PecA was predicted to consist of two aspartic acid residues, the DTG and DSG motifs found at amino acids 293 to 295 and 470 to 472, respectively. These residues are positioned in the center of the putative cleft of the predicted protein structure (Fig. 1B). To confirm whether aspartic protease activity was required for proteolytic processing of PecA, we aimed to create a catalytically inactive site mutant by replacing the conserved aspartic acid residue at position 293 with glycine (PecA_D293G_). This mutated PecA was not cleaved when expressed in the *ΔpecA* strain, demonstrating that the aspartic protease activity of PecA is indeed required for PecA self-cleavage (Fig. 3A).

Interestingly, when the same construct was expressed in wild-type M. marinum, PecA_D293G_ was cleaved, revealing that PecA can be cleaved in *trans* (Fig. 3B). Furthermore, cleaved PecA is present in the Genapol supernatant (GS), but not in the culture supernatant, indicating that PecA is located at the mycobacterial surface (Fig. 3B, GS fraction).

### PecA is directly involved in PE processing.

To confirm that PecA is cleaved by itself and not by another protease, PecA was introduced in E. coli. When expressed in E. coli, PecA generated similarly sized cleavage products as observed in M. marinum. Again, the active site mutant PecA_D293G_ did not produce the specific processed form seen for proteolytic active PecA (Fig. 3C). An additional fragment of lower molecular weight was detected for both PecA and PecA_D293G_, indicating that this fragment was produced by an E. coli protease. To obtain additional proof for self-cleavage of PecA, we performed *in vitro* transcription/translation experiments. Although PecA activity was low under these conditions, we could clearly observe that, also in this cell-free environment, PecA partially cleaved itself, while PecA_D293G_ was not cleaved (Fig. 3D). Taken together, these results demonstrate that PecA is an aspartic protease that can cleave not only LipY but also itself. PecA cleavage is dependent on the conserved aspartic protease residues and can occur in the absence of other mycobacterial proteins.

When we compared the two identified cleavage sites in the linker domain of LipY_tub_, the presence of two consecutive small amino acid (glycine or alanine) residues stood out (Fig. 1D). The variable residues within the YxxxD/E secretion motif of both LipY_tub_ and PecA contain three consecutive alanine residues (YAAAE). In addition, proximal to the secretion motif is a conserved glycine alanine motif at position 84. The presence of consecutive small amino acids within the PE domain combined with the approximately 11-kDa size of the cleaved N-terminal domain led us to hypothesize that the PE domain of PecA and LipY_tub_ is cleaved near or inside the secretion signal motif (17).

To test this hypothesis, we generated *pecA* constructs harboring point mutations near and in the region coding for the PE secretion signal (PecA_A84D_ and PecA_AA88ED_) and studied its processing in M. marinum. The PecA_A84D_ and PecA_AA88ED_ mutants were both partially processed as wild-type PecA (indicated with arrow in Fig. 3E) but differed in size regarding to the secondary products. The secondary product of PecA_A84D_ appeared at full length whereas the secondary product of PecA_AA88ED_ was alternatively processed, shown by an additional band of ∼47 kDa (Fig. 3E and Fig. S4). These data demonstrate that PecA cleaves itself within the PE domain.

10.1128/mBio.01951-19.4 FIG S4 Processing of the PE domain. Immunoblot analysis of M. marinum *ΔpecA* or *ΔpecA* strains expressing PecA_myc_, PecA_A84Dmyc_, PecA_AA88EDmyc_, and PecA_D293Gmyc_ (whole-cell lysate). The arrow indicates the fully cleaved form of PecA. PecA was detected with an anti-Myc antibody. Download FIG S4, PDF file, 0.4 MB. Copyright © 2019 Burggraaf et al. 2019 Burggraaf et al. This content is distributed under the terms of the Creative Commons Attribution 4.0 International license.

### PecA processes PE_PGRS proteins.

**FIG 4:**
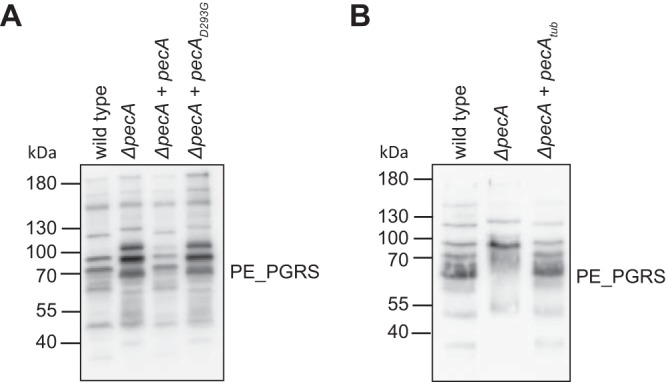
PecA is required for the processing of PE_PGRS proteins. Immunoblotting of whole-cell lysate from M. marinum wild-type M strain, *ΔpecA* strain, and *ΔpecA* strain complemented with M. marinum *pecA_myc_* and *pecA_D293Gmyc_* (A) or M. tuberculosis *pecA_tub_* (B) for endogenous PE_PGRS proteins. PE_PGRS proteins were detected with monoclonal antibodies recognizing the PGRS domain.

With the aid of ESX-5 mutant strains, we previously showed that PecA and LipY are both ESX-5 substrates (7, 23) and belong to the PE_PGRS protein family. Based on the conservation of the PE domains among PE_PGRS proteins and the proteolytic cleavage inside the PE domain of both LipY_tub_ and PecA, we hypothesized that PecA could also cleave other PE_PGRS proteins. We tested this hypothesis by immunoblotting lysates of wild-type and *ΔpecA* strains using a monoclonal antibody directed against the PGRS domain, recognizing various PE_PGRS proteins (7). Although secretion of PE_PGRS proteins seemed to be normal in all strains tested, the PE_PGRS protein pattern of the M. marinum *ΔpecA* mutant appeared different from the wild type (Fig. 4A). Specifically, the *ΔpecA* strain showed several proteins with an increased molecular weight. The different pattern shows an increase of approximately ∼11 kDa for each PE_PGRS protein, corresponding with the size of a PE domain.

This indicates that several or perhaps most PE_PGRS proteins that are secreted under these conditions are dependent on PecA for their processing. Importantly, the PE_PGRS pattern on an immunoblot was restored to wild type when PecA was expressed in the *ΔpecA* strain. Notably, the expression of the active site mutant *pecA_D293G_* did not restore the PGRS phenotype, confirming that the aspartic protease activity is required for proteolytic processing of PE_PGRS proteins.

### M. tuberculosis PecA complements the M. marinum Δ*pecA* mutant.

As mentioned previously, the genome of M. tuberculosis contains a gene (*Rv1983*, *PE_PGRS35*) with high sequence identity (69.6% identical amino acids) and conserved synteny. The identity is especially conserved in the protease domain, implying a similar function. To investigate whether PE_PGRS35 processes PE_PGRS proteins in a similar fashion, we expressed this gene in M. marinum *ΔpecA* and visualized the PE_PGRS proteins by immunoblotting. We observed full complementation, based on the identical PE_PGRS patterns between the complemented strain and the wild-type M. marinum strain (Fig. 4B). We conclude that PE_PGRS35 from M. tuberculosis has proteolytic activity toward PE_PGRS proteins from M. marinum and potentially M. tuberculosis, and therefore, we designate it PecA_tub_.

### PecA is a virulence factor *in vivo*.

PE_PGRS proteins have been implicated to play a role in immune modulation, macrophage invasion, and B-cell responses (9, 27). However, the high similarity between members of this family implies functional redundancy, which hinders their functional resolution. Since PecA processes many of these proteins, including itself, we assessed whether its proteolytic activity contributes to virulence *in vivo*.

We first established that the absence of PecA has no negative effect on replication *in vitro* (Fig. S5A). Next, we monitored intracellular replication in RAW 264.7 macrophages and Acanthamoeba castellanii with mEosFP-expressing wild-type M. marinum and *ΔpecA* strains. Although PecA is responsible for the processing of many PE_PGRS proteins, no or only very small differences in intracellular replication were observed between the wild-type and the *ΔpecA* strains in the phagocytic host cells studied (Fig. S5B to D).

10.1128/mBio.01951-19.5 FIG S5 Effect of PecA on intracellular replication following infection in A. castellanii and RAW 264.7 cells. (A) Growth curves of Mycobacterium marinum M strains. Wild-type, *ΔpecA*, *ΔpecA* plus *pecA_myc_*, or *ΔpecA* plus *ΔpecAD293G_myc_* strains were grown in triplicate in 7H9 supplemented with ADC. OD was measured at indicated time points and plotted against time. (B and C) A. castellanii cells were infected with mEosFP-expressing wild-type and *ΔpecA* M. marinum M strains. Number of infected cells was determined at 4 and 24 hours postinfection by flow cytometry (B, gating strategy; C, bar graph of all data points). (D) RAW 264.7 cells were infected with wild-type and *ΔpecA* M. marinum M strains containing mEosFP. The number of infected cells was determined 0, 20, and 43 hours postinfection by flow cytometry. Data are presented as mean ± SD values (*n* = 3) (10.000 cells per sample). Download FIG S5, PDF file, 0.5 MB. Copyright © 2019 Burggraaf et al. 2019 Burggraaf et al. This content is distributed under the terms of the Creative Commons Attribution 4.0 International license.

**FIG 5:**
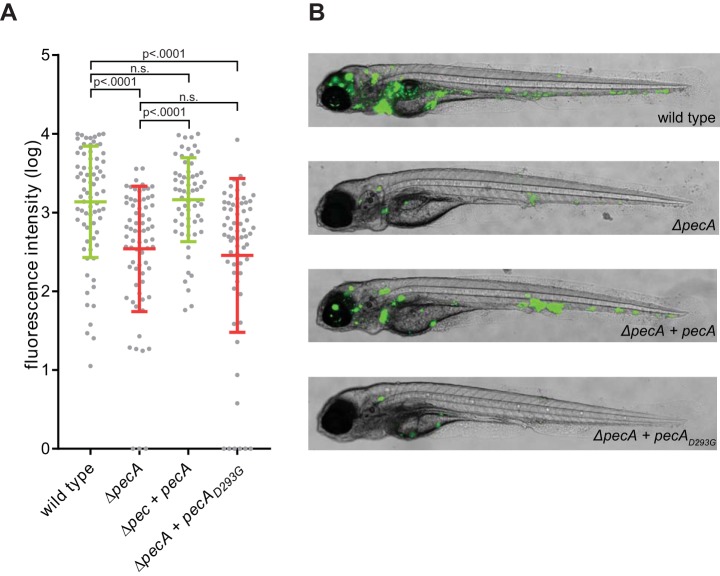
Deletion of PecA causes attenuation of virulence in an *in vivo* zebrafish infection model. (A) A total of 273 zebrafish larvae were infected 1 day postfertilization (dpf) with wild-type (75 larvae), *ΔpecA* (71 larvae), *ΔpecA* plus *pecA_myc_* (62 larvae), or *ΔpecA* plus *ΔpecA_D293Gmyc_* (65 larvae) M. marinum M strains (see Table S2 for input CFU). Graph shows relative infection from 4 independent experiments as calculated by normalized integrated fluorescent intensity per larva at 4 days postinfection, with mean intensity and standard deviation for the entire population. Similar effects were obtained for CFU counts at 4 dpi with wild-type, *ΔpecA*, and *ΔpecA* plus *pecA_myc_* strains (Fig. S6). (B) Representative images of infected larvae are shown for each strain.

To assess the role of PecA in an animal model, zebrafish larvae were infected with M. marinum wild-type and *ΔpecA* strains. In contrast to the experiments with phagocytic cells, the *ΔpecA* strain showed reduced outgrowth (5-fold change) after 4 days compared to wild-type bacteria, when measured by fluorescence microscopy (Fig. 5A and B) and CFU plating (Fig. S6). This phenotype was fully complemented by reintroducing *pecA* in the deletion mutant when measured by fluorescence microscopy and partially when measured by CFU counting. Importantly, the active site mutant PecA_D293G_ failed to complement the *ΔpecA* phenotype, demonstrating that proteolytic activity of PecA is directly contributing to virulence *in vivo*.

10.1128/mBio.01951-19.6 FIG S6 Deletion of *pecA* causes attenuation in an *in vivo* zebrafish infection model. Zebrafish larvae were infected 1 day after fertilization with wild-type (4 CFU), *ΔpecA* (4 CFU), or *ΔpecA* plus *pecA_myc_* (9 CFU) M. marinum M strains. Graphs show absolute infection as counted by CFU per larva at 4 days postinfection. Data are presented as mean ± SD, and the 1-way ANOVA was used for statistical analysis on the log-transformed CFU data. The *P* value did not reach statistical significance when comparing CFU counts between the *ΔpecA* and *ΔpecA* plus *pecA_myc_* strains (*P* = 0.0944). Download FIG S6, PDF file, 0.4 MB. Copyright © 2019 Burggraaf et al. 2019 Burggraaf et al. This content is distributed under the terms of the Creative Commons Attribution 4.0 International license.

10.1128/mBio.01951-19.8 TABLE S2 Input CFU and number of larvae per experiment per strain. One nl was injected into the larvae. Pre, number of bacteria present in inoculum per nanoliter prior to injecting larvae. Post, number of bacteria present in inoculum per nanoliter after injecting larvae. n.d., not done; strains were not yet generated. Download Table S2, PDF file, 0.1 MB. Copyright © 2019 Burggraaf et al. 2019 Burggraaf et al. This content is distributed under the terms of the Creative Commons Attribution 4.0 International license.

## DISCUSSION

**FIG 6:**
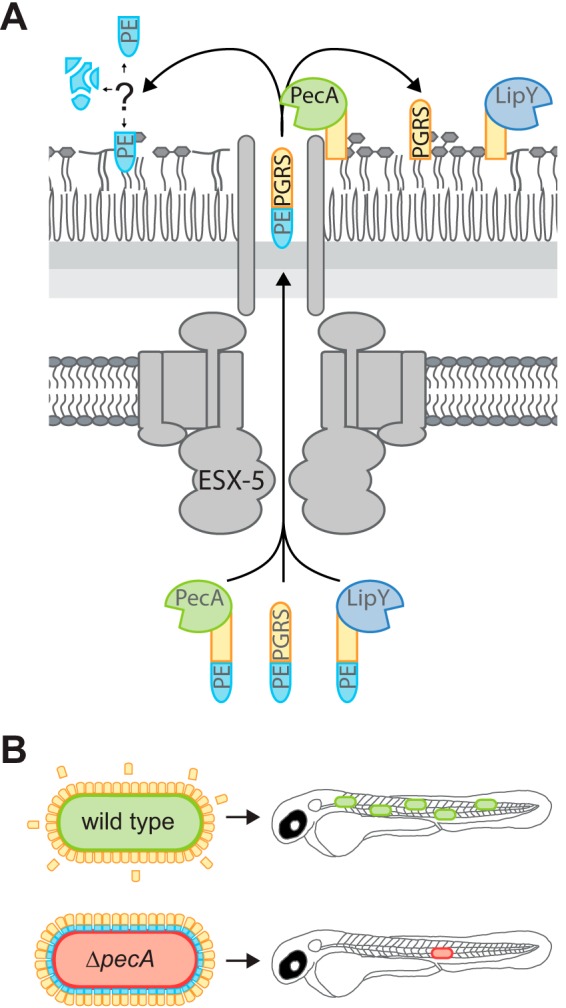
Model: PecA processes PE-PGRS proteins and is important for virulence. (A) PecA removes the PE domain of PE-PGRS proteins, including LipY_tub_ and itself, at the surface of Mycobacterium marinum. The processed PE_PGRS proteins might be of importance during infection, although their exact role, as well as the fate of the PE domains, remains unknown. (B) Proteolytic activity of PecA is suggested to play a role in virulence of M. marinum, as was observed during infection in zebrafish larvae.

For most bacterial pathogens, secreted and surface-associated proteins play a crucial role in host-pathogen interactions. The largest families of secreted proteins in pathogenic mycobacteria are the PE and PPE protein families, whose role in host-pathogen interactions remains enigmatic (9). A large subset of the PE proteins belongs to the PE_PGRS family and is secreted by the ESX-5 system (7, 8). PE_PGRS proteins have a conserved N-terminal PE domain (100 amino acids [aa], ∼11 kDa) containing the YxxxD/E secretion motif and a larger C-terminal domain that is characterized by GGAGGX repeats. In this study, we have provided new mechanistic insights into the processing of M. marinum-secreted PE_PGRS proteins by PecA, a PE_PGRS protein with aspartic protease activity (Fig. 6).

Previously, Barathy and Suguna (25) resolved the structure of the C-terminal domain of PE_PGRS16 (Rv0977) and showed that it has an aspartic protease fold. In the same study, they described two other PE proteins of M. tuberculosis that could have aspartic protease activity: PE26 (Rv2519) and PE_PGRS35 (Rv1983, PecA_tub_). All three proteins contain the DTG/DSG sequences that are characteristic for the catalytic domain of aspartic proteases. Whereas this previous study was unable to demonstrate proteolytic activity for PE_PGRS16, in our study we conclusively show that PecA is an active protease that is essential to process PE_PGRS proteins at the cell surface.

LipY_tub_, one of the PecA substrates, contains at least three different processing sites, two in the linker domain and one near the end of the PE domain. This extensive processing could be an exception, since we observe an apparent molecular weight difference consistent with the removal of the PE domain (or part thereof) for PecA and other PE_PGRS proteins. Mutations near or inside the secretion signal motif YxxxD/E of PecA resulted in reduced and even altered processing of the PE domain, demonstrating that the PE domain is cleaved by PecA. We were, however, unable to create a mutant that fully blocked processing by PecA. This is not unexpected, since cleavage sites of aspartic proteases, such as HIV-1 protease, are known to be difficult to predict. The HIV-1 protease cleavage sites not only rely on sequence but also are determined by local structure and physicochemical features of the cleavage site (28).

As mentioned above, the processing of other PE_PGRS proteins also seems to indicate the removal of a PE domain-sized fragment. However, some caution is warranted since we did not confirm the identity of the PE_PGRS proteins visualized by Western blotting. Whether the PE domain remains intact after cleavage and associated with the bacterium to fulfill a function beyond enabling secretion of the C-terminal domain remains a question to be answered.

We found evidence that processing of PE proteins takes place at the cell surface. First, PecA itself is an ESX-5 substrate, since the culture filtrate of an *esx-5* mutant of M. marinum lacks PecA (MMAR_2933), as Abdallah et al. demonstrated by mass spectrometry (MS) analysis (7). Second, processing of LipY does not affect its surface localization and lipase activity. This last result is in sharp contrast with results obtained with recombinant LipY, which showed increased lipase activity upon removal of the PE domain (22, 26). To reconcile these observed differences, we speculate that the PE domain is interacting with (glyco)lipids in the mycobacterial membrane, preventing inhibition of lipase activity. However, prior to secretion by the ESX-5 system, the PE domain could be inhibiting LipY to prevent undesired intracellular lipase activity. Similarly, PecA exhibited strongly reduced protease activity, measured by self-cleavage, when expressed in E. coli or *in vitro.* This suggest that its natural surrounding at the cell surface is important for optimal activity. Improper folding could be an alternative explanation for the observed reduction in enzymatic activities of both LipY and PecA when produced in E. coli.

Although no obvious difference in intracellular growth was observed in phagocytic cells, deletion of *pecA* in M. marinum resulted in moderate attenuation in the zebrafish larva infection model. This phenotype was restored by introducing the wild-type gene coding for PecA but not by the inactive PecA_D293G_, indicating that substrate processing affects their biological function. It would be interesting to identify which PE or PE_PGRS proteins are required to be cleaved to function as a virulence factor. However, this is challenging, since there are as many as 110 PE_PGRS proteins in M. marinum (63 in M. tuberculosis), and the exact role of each PE_PGRS proteins during infection is unknown. Previously, we have demonstrated that PE_PGRS proteins reduce virulence, since strains lacking PE_PGRS (and PPE-MPTR) secretion, exhibited increased virulence (16). These contradictory results could be explained by the possibility that PecA might have other substrates in addition to PE_PGRS proteins that are involved in virulence. Follow-up research has to reveal whether PecA substrate specificity goes beyond the PE_PGRS proteins or even beyond ESX-5 substrates. By looking into the substrate specificity and the role of these different substrates, we will further elucidate the role of PE_PGRS proteins and PecA during infection.

PecA from M. marinum shares 69.6% identity with PecA from M. tuberculosis. And indeed, we showed that a *pecA* mutant in M. marinum was complemented with *pecA_tub_*. It is tentative to speculate that PecA_tub_ plays a similar function in virulence as PecA from M. marinum. However, possibly PecA in M. tuberculosis has (partial) redundancy with the two homologs, i.e., PE26 and PE_PGRS16.

Next to the attenuation *in vivo*, the importance of PecA in pathogenic mycobacteria can be illustrated by its high degree of conservation in the mycobacterial genome. Most PE_PGRS proteins show high variability between clinical M. tuberculosis strains and accumulate mutations faster than expected (29). In contrast, PecA is one of the most conserved PE_PGRS proteins. The mutation rate of PecA is even lower than the average mutation rate of the entire genome, again suggesting an important and conserved role for this protease (30).

Taken together, we show that PecA is a unique aspartic protease, in respect to its bacterial origin and extracellular localization. PecA is required for the processing of many PE_PGRS proteins, the largest family of secreted proteins in pathogenic mycobacteria. Future investigations will have to reveal whether PecA-dependent processing is limited to PE_PGRS proteins and will address the role of PecA and its two homologs in M. tuberculosis.

## MATERIALS AND METHODS

### Bacterial strains and growth conditions.

Mycobacterium marinum E11 (31) and M (32) were grown on 7H10 agar plates supplemented with Middlebrook oleic acid-albumin-dextrose-catalase (OADC) or in 7H9 broth (Difco-BD Biosciences) supplemented with Middlebrook ADC and 0.05% Tween 80 (Sigma) at 30°C with shaking at 90 rpm. M. tuberculosis CDC1551 was grown in 7H9 supplemented with Middlebrook ADC and 0.05% Tween 80 (Sigma) at 37°C.

Escherichia coli DH5α and E. coli BL21 were grown in LB medium (Difco-BD Biosciences) at 37°C with shaking at 200 rpm.

For all cultures, hygromycin was used at a concentration of 50 μg/ml, kanamycin was used at 25 μg/ml, and streptomycin was used at 30 μg/ml.

### Construction of *pecA* deletion mutant.

M. marinum *ΔpecA* was constructed using the mycobacteriophage method developed by the Jacobs laboratory (33) and described in reference 12. Briefly, *MMAR_2933* was deleted by synthesizing fragments of flanking regions of *MMAR_2933* by PCR (primer set MMAR_2933 LF and MMAR_2933 LR for the 5′ region and primer set MMAR_2933 RF and MMAR_2933 RR for the 3′ region; primers are listed in Table S1 in the supplemental material). Resulting amplicons were digested with AlwNI and DraIII, cloned into the Van91I-digested p0004s plasmid containing a hygromycin resistance cassette and the *sacB* gene to enable selection on sucrose sensitivity, introduced to the PacI site of phasmid phAE159, and electroporated into Mycobacterium smegmatis mc^2^155 according to the method of Bardarov et al. (34). The phage was isolated in high titers and incubated with M. marinum strain M. Colonies were selected on hygromycin plates and verified for sucrose sensitivity. The deletion was confirmed with PCR and sequencing.

10.1128/mBio.01951-19.7 TABLE S1 Primers used in this study. Overview of the primes used in this study. For site-directed mutagenesis, indicated primers were used in combination with primers containing the reverse-complement sequence. Download Table S1, PDF file, 0.1 MB. Copyright © 2019 Burggraaf et al. 2019 Burggraaf et al. This content is distributed under the terms of the Creative Commons Attribution 4.0 International license.

### Plasmid construction.

The secretion-optimized synthetic LipY_tub_ construct, named pSMT3-*lipY-OVA_2_*, contains amino acids 1 to 98 and 201 to 205 from LipY_tub_ fused to a fragment of chick ovalbumin (amino acids 259 to 357). This construct was identified by screening for enhanced secretion after random mutagenesis of LipY_tub_-1–205 fused to chick ovalbumin (amino acids 259 to 357) in the pSMT3 plasmid. This work is described in the work of Burggraaf et al. (24).

To create pMV361*-pecA*, pMV361-*eccB_5_-mycP_5_-HA* (described by reference 35) was restricted with EcoRI and HindIII. MMARE11_28540 (*MMAR_2933*, *pecA*) was amplified by PCR using primers PecA-pMV-Fw and PecA-pMV-Rv (primers are listed in Table S1) and subsequently inserted by In-Fusion cloning (TaKaRa Bio). After restriction with XhoI and HindIII to introduce an annealed oligonucleotide containing the Myc tag, the mutations D293G, A84D, and AA88ED were introduced using site-directed mutagenesis (Agilent Technologies). pST5552-pecA_tub_ was created by restriction cloning. PecA_tub_ was PCR amplified from genomic H37Rv DNA with the primers Rv1983-Fw and Rv1983-Rv. The PCR product was digested with EcoRI and XhoI and ligated into the pST5552 plasmid (a pMV261-based vector with a theophylline-inducible riboswitch) digested with EcoRI and SalI. The plasmids were verified with sequencing.

To enable *in vitro* transcription, pET28A-*iCAP* was restricted with XhoI and NcoI. *pecA* and *pecA_D293G_* were amplified by PCR from pMV-*pecA* and pMV-*pecA_D293G_*, respectively, using primers PecA-pET-Fw and PecA-pET-Rv and introduced in pET28A (NcoI/XhoI) by infusion cloning.

Plasmids were introduced to M. marinum by electroporation and to E. coli DH5α by transformation according to standard heat shock protocol.

### Transposon mutagenesis and double filter screening.

A transposon library was generated for strain M. marinum E11 transformed with pSMT3-*lipY-OVA_2_* (published in reference 24) using the mycobacterium-specific phage phiMycoMarT7 containing mariner-like transposon Himar1 (36).

After transposon mutagenesis, M. marinum pSMT3-*lipY-OVA_2_* was grown on a nitrocellulose membrane (0.45 μm; Merck Millipore) placed on a 7H10 plate supplemented with Middlebrook OADC (Difco-BD Biosciences). Plates were incubated at 30°C. When colonies formed, the membrane was transferred onto a new membrane already placed on a fresh plate and incubated overnight at 30°C. The second membrane was incubated with antibodies directed against the influenza virus hemagglutinin epitope (HA.11; Covance) and the secondary antibody horseradish peroxidase-conjugated goat anti-mouse IgG (American Qualex Antibodies). Signal was detected with enhanced chemiluminescence (ECL) Western blotting detection reagent (Amersham Bioscience). Selected transposon mutants and complemented strains were grown to an optical density at 600 nm (OD_600_) of 0.8 to 1.2 in 7H9 supplemented with Middlebrook ADC (Difco Biosciences), 10-μl drops were placed on the nitrocellulose filter on a 7H10 plate, and the procedure for analyzing secretion was repeated.

### *In vitro* transcription and translation.

pET28A-*pecA* and pET28A-*pecA_D293G_* were expressed with the PURExpress *in vitro* protein synthesis kit (New England Biolabs [NEB]) according to the manufacturer’s protocol. Briefly, 100 ng plasmid DNA was added to a mixture of solutions A and B, supplemented with RNase inhibitor (Thermo Scientific). The reaction mixture was incubated for 2 h at 37°C and subsequently put on ice for 15 min. Solubilization/denaturation (SD) buffer (containing 100 mM dithiothreitol and 2% SDS) was added before loading samples on the gel for SDS-PAGE.

### Secretion analysis.

One day prior to making samples, cultures were washed with 7H9-0.2% dextrose-0.2% glycerol-0.05% Tween 80 to remove ADC. Cultures were grown in this medium at 30°C, with shaking at 90 rpm to an OD_600_ of 0.8 to 1.2. Cultures were harvested, and the supernatant was filtered through an 0.2-μm Millipore filter and precipitated with 10% trichloric acetic acid (Sigma) and 36 μg/ml tRNA (Roche). Pellets were washed with phosphate-buffered saline (PBS). Two OD units was taken and dissolved in SD buffer to a concentration of 0.02 OD/μl, making the pellet sample. Two OD units was incubated with 0.5% Genapol X-080 (in PBS) for 30 min at room temperature (RT). After spinning down, the supernatant was taken and diluted with SD buffer to a final concentration of 0.016 OD/μl, forming the Genapol supernatant fraction. The remaining pellet was washed with PBS and dissolved in SD buffer to a concentration of 0.02 OD/μl, forming the Genapol pellet fraction. Pellet samples were sonicated. All samples were incubated 5 min at 98°C.

### SDS-PAGE and immunoblotting.

Samples were loaded on a 12.5% SDS-PAGE gel. Subsequently, proteins were transferred to nitrocellulose membranes for Western blotting. Blots were blocked with 5% skimmed milk in PBS and incubated with antibodies recognizing the influenza virus hemagglutinin tag (HA.11; Covance), GroEL2 (CCs44; J. Belisle, NIH, Bethesda, MD, USA), PGRS domain (7), or the Myc tag (dMyc; Abcam). Secondary antibodies used were horseradish peroxidase conjugated to goat anti-mouse IgG or to goat anti-rabbit (Rockland). Secondary antibodies were detected with chemiluminescence (ECL).

### Flow cytometry analysis.

Bacteria were grown to an OD_600_ of 0.8 to 1.2 in 7H9 with ADC and without Tween 80. Bacteria were pelleted, washed with PBS with 0.5% bovine serum albumin (BSA; Sigma), and incubated for 1 h with antibodies recognizing the influenza virus hemagglutinin tag (HA.11; Covance). After washing with PBS with 0.5% BSA, bacteria were incubated with secondary goat anti-mouse IgG conjugated to Alexa 647 antibodies for 1 h. After washing with PBS with 0.5% BSA, bacteria were analyzed by flow cytometry (Attune NxT; ThermoFisher). As a control, bacteria were incubated only with the secondary antibodies.

### Bioinformatic analysis.

Data obtained with the lipase assay were analyzed with an unpaired *t* test. The fluorescent intensity data obtained from the zebrafish experiments were normalized and log_10_ transformed to obtain a Gaussian distribution. Normalization was performed by subtracting the geometric mean of uninfected larvae and setting the maximum value for each experiment arbitrarily to 10,000. Larvae with a negative fluorescence were set to 1 to allow for log_10_ transformation. A 1-way analysis of variance (ANOVA) was performed on the transformed data and on the CFU data to test the null hypothesis: there is no difference between the groups and equality between means. When statistical significance (*P* < 0.05) is reached, the *P* value is specified.

### Structure prediction.

The homology model of the protease domain of PecA was obtained using RaptorX (37) and the crystal structure of the protease domain of PE_PGRS16 (PDB identifier [ID] 4EHC) as a template (25). The model was refined using ModRefiner (38). The model was adjusted to remove clashes using Coot (39) and energy minimized using Phenix (40).

### N-terminal sequencing.

Bacterial cultures were washed with 7H9-0.2% dextrose-0.2% glycerol and grown to an OD_600_ of 0.8 to 1.0. Bacterial pellets were incubated with 0.5% Genapol X-080 for 30 min at RT. The supernatant was collected and precipitated with 10% trichloroacetic acid (TCA) and 29 μg/ml tRNA. Samples were incubated 1 h on ice, before being washed with acetone. The supernatant was discarded, and pellets were dried at RT. Pellets were dissolved in SD buffer and incubated for 5 min at 98°C.

Samples were run on a 12.5% SDS-PAGE gel supplemented with 0.4 mM gluconic acid (Merck). The gel was blotted on a polyvinylidene difluoride (PVDF) membrane (Bio-Rad). The blot was stained with 0.1% Coomassie brilliant blue R250, 50% methanol, 1% acetic acid, and destained with 50% methanol. The band corresponding to the cleaved product of LipY_A150D_ was excised, and the 5 N-terminal amino acids were analyzed by Edman N-terminal sequencing (Alphalyse, Odense, Denmark).

### Lipase activity assay.

Bacteria were grown to mid-log phase in 7H9 medium (Difco) supplemented with 10% ADC (BD Bioscience) with 0.05% Tween 80 and diluted to an OD_600_ of 0.2. One hundred sixty microliters of bacterial suspension was mixed with 25 μl development solution DGGR [10 mg/ml BSA, 120 mM NaCl, 50 mM Tris, pH 7.5, 1% Triton X-100, 4 μM 1,2-di-*O*-lauryl-*rac*-glycero-3-(glutaric acid 6-methylresorufin ester)]. Fluorescence was measured in a plate reader at an excitation of 530 nm and emission of 600 nm. Paraoxon (POX; 10 μM; Sigma) is a lipase inhibitor, used as a control for inhibition of LipY lipase activity.

### Zebrafish larva infection procedure.

Transparent casper zebrafish larvae were removed from their chorion with tweezers and infected at 1 day postfertilization (dpf) via the caudal vein with a bacterial suspension. The injected CFU was determined before and after injection of the larvae to rule out blockage of the needle (see Table S2 in the supplemental material). Injection stocks were prepared by growing bacteria until the logarithmic phase (OD_600_ of 0.7 to 1). Bacteria were spun down at low speed for 1 min to remove the largest clumps, washed with 0.3% Tween 80 in phosphate-buffered saline (PBS), and sonicated briefly for declumping. Bacteria were resuspended in PBS with 20% glycerol and 2% polyvinylpyrrolidone and stored at −80°C. Before use, bacteria were resuspended in PBS containing 0.17% (vol/vol) phenol red (Sigma) to aid visualization of the injection process. To determine the exact number of bacteria injected, the injection volume was plated on 7H10 plates containing the proper antibiotic selection. At 4 days postinfection (dpi), larvae were analyzed with an Olympus IX83 fluorescence microscope. Bright-field and fluorescence images were acquired with an Olympus IX83 microscope equipped with an Orca-flash 4.0 LT camera. Infection levels were quantified with CellProfiler 3.15. Zebrafish were manually delineated followed by integrating the fluorescent intensity of the entire fish.

### CFU counting.

Viable bacterial counts present in whole larvae were determined by plating larva homogenates, decontaminated with BBL MycoPrep (BD), on Middlebrook 7H10 agar.
