# Supplementary material for: Type VII Secretion Substrates of Pathogenic Mycobacteria Are Processed by a Surface Protease
Source: mBio. 2019 Oct 29;10(5):e01951-19. doi: 10.1128/mBio.01951-19 (PMC6819658; doi:10.1128/mBio.01951-19)
Supplement: FIG S1 [file mBio.01951-19-sf001.pdf]

S1A

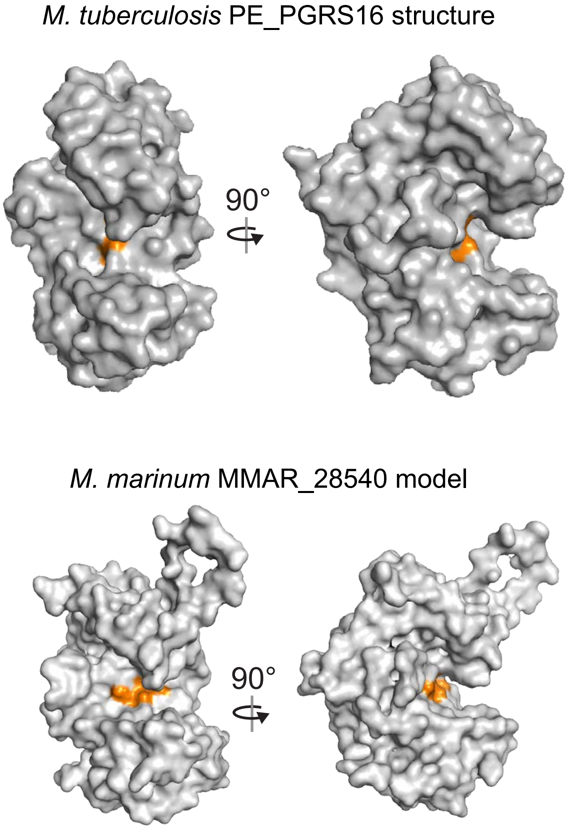
M. tuberculosis PE_PGRS16 structure
90°
M. marinum MMAR_28540 model
90°

Figure S1B

```
PGA3                           WKAPTLVDEQPLENYLDMEYFGTIGI-GTPAQDFTVVFDTGSSNLWVPS-------VYCS
HIV-PR                         -----------PQVTLWQRPLVTIKI-GGQLKE--ALLDTGADDTV-----------LEE
PE_PGRS35(Rv1983)              -----DGRTVPLEIIHVTEPTVHANVNGGPTST--ILVDTGSAGLVVSPEDVGGILGVLH
MMARE11_28540(MMARE_2933)      --------TVPLEVVNVTEPVVNVNVNGGHSTP--VLIDTGSAGLVMQVKDVGGPLGLLR
MMAR_2272                      -------------------PVVTISVGGGPGIA--VTVDTGASGLLVRPQDV----NLQS
PE_PGRS16(Rv0977)              -STTLTNATVPLQLVNTTEPVVFISLNGGQMVP--VLLDTGSTGLVMDS-QF----LTQN
PE26(Rv2519)                   ---DPVNVAVPLRVENNF-PLVNLLVNRGPTVP--ILLDTGSSSLVIPFWKI----GWQN
MMAR_1538                      ------NASVPLYLDNNF-PAVNVSINGGPSVP--VLLDTGSAGLVVPIWDI----GLQN
                                                        :           .***: .

PGA3                           SLACTNHNRFNPEDSS----TYQSTSETVSITYGTGSMTGILGYDTVQVGGISDTNQIFG
HIV-PR                         MSLP-GRWKPKMIGGI------------------GGFIK-----------VRQYDQILIE
PE_PGRS35(Rv1983)              MGLPTGLSISGYSGGL----YYIFATYTTTVDFGNGIVTAPTA---VNVVLLSIPTSPFA
MMARE11_28540(MMARE_2933)      MGLPSGISMSAYSGGL----TYLFATYPTTVDFGNGIVTSTTG---VDVVLFSIPTSPYA
MMAR_2272                      LGTPTGSGAVTYGNSSYAFNTVQYQTYQTTVNFGNGIVTNPTN---VAVATSATQT-ING
PE_PGRS16(Rv0977)              FGPVIGTGTAGYAGGL----TYNYNTYSTTVDFGNGLLTLPTS---VNVVTSSSPGTL--
PE26(Rv2519)                   LGLPTGFDVVHYGNGV----SIVYADVPTTVDFGGGAATTPTS---VHVGILPYPRNLDS
MMAR_1538                      LGVPTGFDVIRYGNGV----NILYADFNTTVDFGSGAVTAPTS---VQVGILPFPTSLQG
                                    .       ..                    *  .

PGA3                           LS-----------ETEPGSFLYYAPFDGILGLAYPSISS---SGATPVFDNIWNQGLVSQ
HIV-PR                         IC-------------------------GHKAIG-TVLVG---PTPVNII-----------
PE_PGRS35(Rv1983)              ISTYFSALLADPTTTPFEAYFGAVGVDGVLGVG-PNAVG---PGP-SIPTMALPGDLNQG
MMARE11_28540(MMARE_2933)      LTTWLNALWSNPLTTPFDAYFQSAGVDGVLGVG-PNAVG---PGP-SIPTQALGGGLGQG
MMAR_2272                      VT-----------TSIPLSSLPL-----YLGIG-PNNDF---PLP-DQVTAALPGDLNQG
PE_PGRS16(Rv0977)              -----------------GNFLSRSGAVGVLGIG-PNNGF---PGTSSIV-TAMPGLLNNG
PE26(Rv2519)                   LV-----------LIASGGAFGPNG-NGILGIG-PNVGSYAVSGPGNVVTTDLPGQLNEG
MMAR_1538                      LT-----------LIAMGNGFGPTG-HGVLGIG-PNINA-ATGGHGNVVTTALPGQLNEG
                                                             .:. .

PGA3                           DLFSVYLSADDQSGSVVIFGGIDSSYYTGSLNWVPVTVEGYWQITVDSITMNGEAIACAE
HIV-PR                         -------------------GRNLLTQIGCTLNFPQVTL---WQRPLVTIKIGGQLKE---
PE_PGRS35(Rv1983)              VLI-------DAPAGELVFGPNPLPAPNVEVVGSPITT---LYVKID----GGTPIPVP-
MMARE11_28540(MMARE_2933)      LLI-------DMKGGELVFGPNPL-TPEFSISGAPIAT---LWVSVN----GGAPVAVP-
MMAR_2272                      VLI-------NTNLGYLQFGANPL-TPVASVTGSPVTD---LQIQIN----NGPLQPATG
PE_PGRS16(Rv0977)              VLI-------DESAGILQFGPNTL-TGGITISGAPIST---VAVQID----NGPLQQAP-
PE26(Rv2519)                   TLI-------DIPGGYMQFGPNTG-TPITSVTGAPITV---LNVQIGGYDPNGGYWSLP-
MMAR_1538                      ELI-------NIPQGYMQFGPNTG-TPITSVSGVPITT---LDVQFGGYDPLGTYYPVT-
                                                  *          :    ::        .      *

PGA3                           GCQAIVDTGTSLLTGPTSPIANIQSDIGASENSD-GDMVVSCSAISSLPDIVFTINGVQY
HIV-PR                         ---ALLDTGADDTVLEEMSLP----GRWKPKMIGGIGGFIKVRQYDQILIEICGHKAIGT
PE_PGRS35(Rv1983)              ---SIIDSGGVTGTIPSYVIG----SGTLP-----ANTNIEVYTSPG-GDRLYAFNTNDY
MMARE11_28540(MMARE_2933)      ---SIIDSGGVMGTIPSSVIG----GSTLP-----ANTNITVYTDNTMTTEVYHYSTNDY
MMAR_2272                      ---SFIDSGGLYGTIPSSLIPGVPVGYSVP-----VGTTITVYTTDG--VQLYSQTVTGS
PE_PGRS16(Rv0977)              ---VMFDSGGINGTIPSA-LASLPSGGFVP-----AGTTISVYTSDG-QTLLYSYTTTAT
PE26(Rv2519)                   ---SIFDSGGNHGTLPAVILGTGQTTGYAP-----PGTVISISIHDN-QTLLYQYTTTAS
MMAR_1538                      ---SIVDSGGNHGTIPGIILGTGQTSGVVP-----PGTVISISTNDN-QTLLYSYTTTGT
                                   :.*:*    .     :         .      .  :           :   .

PGA3                           PVPPSAYILQSEGSCISGFQGMNLPTESGELWILGDVFIRQYFTVFDRANNQVGLAPVA-
HIV-PR                         -------VLVGPTPVN--------------------IIGR---------NLLTQIGCT-L
PE_PGRS35(Rv1983)              R-P----TVISSGLMNTGF----LP------FRFQPVYID---------YSPSGIGTTVF
MMARE11_28540(MMARE_2933)      Q-P----TVISSGLMNTGF----LP------FWNQPVYID---------YSPAGTGTTVF
MMAR_2272                      TNAPL--VVPSNNPFNTGN----YP------FLLGPIYIS---------NSPTGGGQTIF
PE_PGRS16(Rv0977)              NTP----FVTSGGVMNTGH----VP------FAQQPIYVS---------YSPTAIGTTTF
PE26(Rv2519)                   NSP----VVTADPRLNTGL----TP------FLLGPVYIS---------NNPSGVGTVVF
MMAR_1538                      DSP----VVTGNIPMNTGL----LP------FALGPVYIS---------NSPSGVGTVVF
                                       : .                         :                  . .

PGA3                           -----
HIV-PR                         NF---
PE_PGRS35(Rv1983)              DHPA-
MMARE11_28540(MMARE_2933)      DMP--
MMAR_2272                      DF---
PE_PGRS16(Rv0977)              N----
PE26(Rv2519)                   NYPPP
MMAR_1538                      NYPPP
```
