# Supplementary figures and images for: Type VII Secretion Substrates of Pathogenic Mycobacteria Are Processed by a Surface Protease
Source: mBio. 2019 Oct 29;10(5):e01951-19. doi: 10.1128/mBio.01951-19 (PMC6819658; doi:10.1128/mBio.01951-19)

# S2

kDa

wild type

LipY<sub>A150D</sub>

70

55

40

35

25

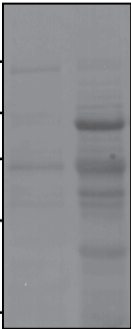

Supplement: FIG S2 [file mBio.01951-19-sf002.pdf]

# S3

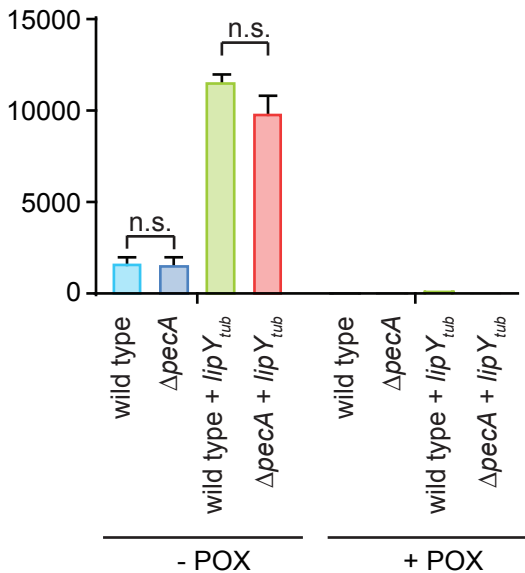

Supplement: FIG S3 [file mBio.01951-19-sf003.pdf]

# S4

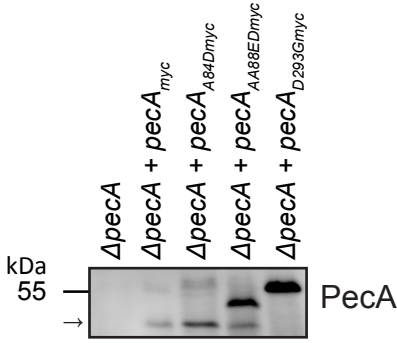

Supplement: FIG S4 [file mBio.01951-19-sf004.pdf]

**S5****A**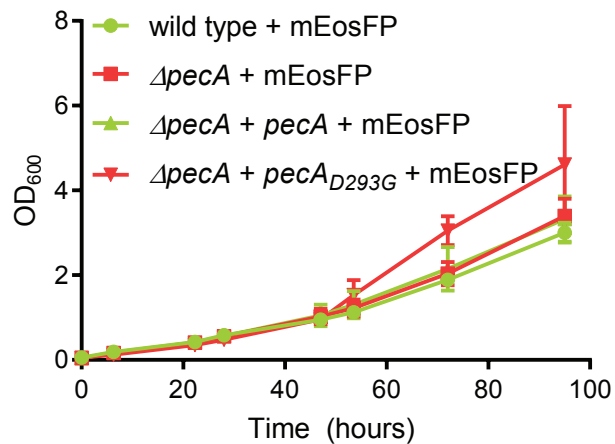**B**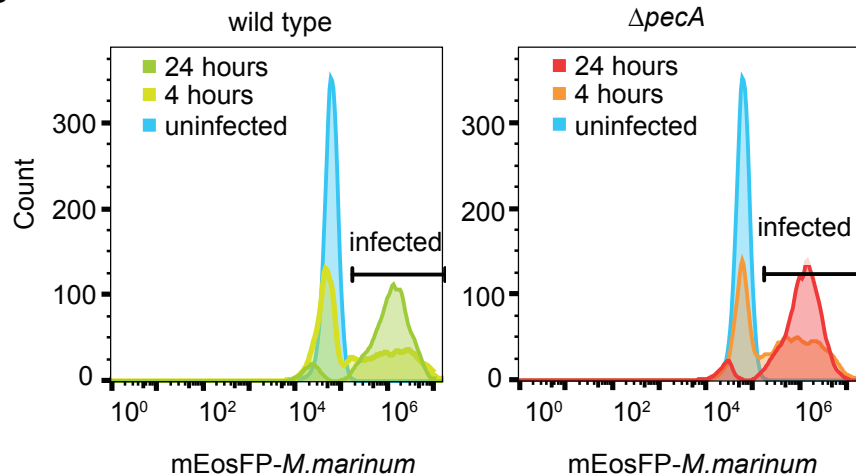**C**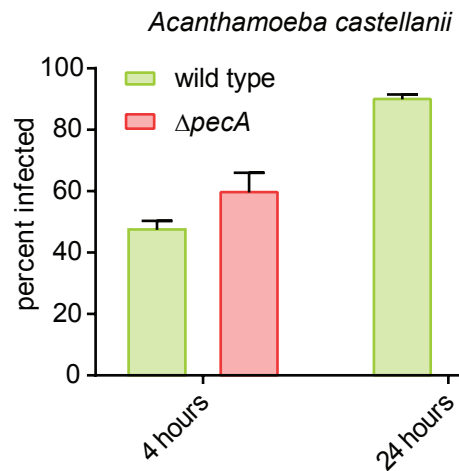**D**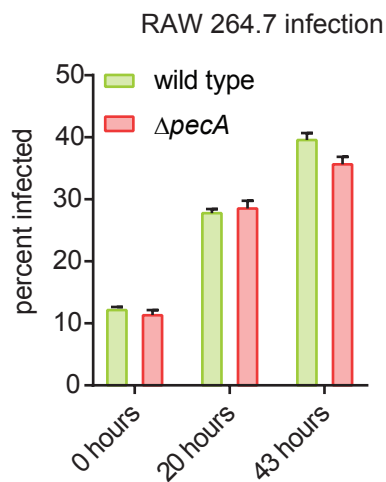

Supplement: FIG S5 [file mBio.01951-19-sf005.pdf]

# S6

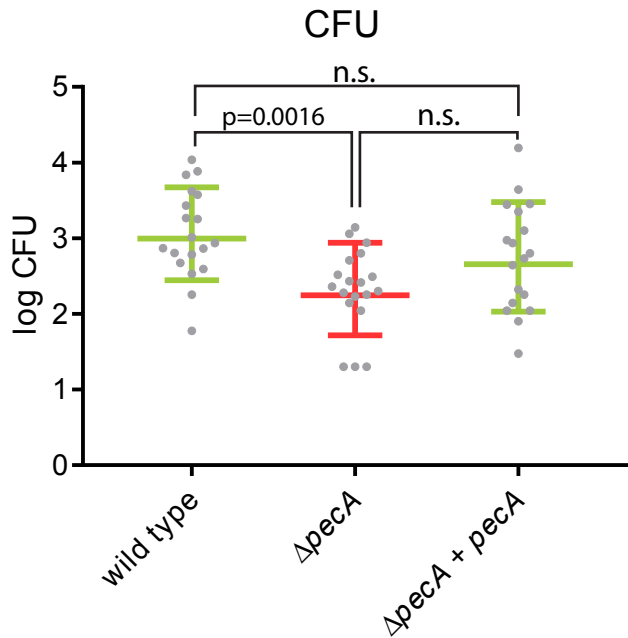

Supplement: FIG S6 [file mBio.01951-19-sf006.pdf]
