# Supplementary material for: Type VII Secretion Substrates of Pathogenic Mycobacteria Are Processed by a Surface Protease
Source: mBio. 2019 Oct 29;10(5):e01951-19. doi: 10.1128/mBio.01951-19 (PMC6819658; doi:10.1128/mBio.01951-19)
Supplement: TABLE S2 [file mBio.01951-19-st002.pdf]

Table S2

	wild type			*ΔpecA*			*ΔpecA+pecA*			*ΔpecA+pecA $_{D293G}$*		
experiment #	# of larvae	pre	post	# of larvae	pre	post	# of larvae	pre	post	# of larvae	pre	post
1	11	73	80	8	42	39	n.d.	n.d.	n.d.	n.d.	n.d.	n.d.
2	20	2	6	20	5	2	18	12	6	19	4	7
3	22	196	145	21	121	83	20	241	190	23	102	89
4	22	20	17	22	38	30	24	24	24	23	39	52
Total larvae	75			71			62			65		
