# Supplementary material for: Type VII Secretion Substrates of Pathogenic Mycobacteria Are Processed by a Surface Protease
Source: mBio. 2019 Oct 29;10(5):e01951-19. doi: 10.1128/mBio.01951-19 (PMC6819658; doi:10.1128/mBio.01951-19)
Supplement: TABLE S1 [file mBio.01951-19-st001.pdf]

Table S1

Primer name	Primer sequence 5' → 3'
PecA-pMV-Fw	CCGGAGGAAAGAATTCCATGTGTCGCTTCTGGTGGTG
PecA-pMV-Rv	CGACATCGATAAGCTTCTAAGGGTTGTTGAAGACCGTTGTG
Rv1983-Fw	AAAGAATTCCATGTGTCATTTCTGGTCGTGGT
Rv1983-Rv	TTTCTCGAGGGATGATCAAAGACTGTTGTCCC
PecA-pET-Fw	AGGAGATATACCATGTCGCTTCTGGTGGTGG
PecA-pET-Rv	GGTGGTGGTGCTCGAGCAAGTCCTCTTCCGAAATCAGCTTT
PecA-A89D	TCGGCCGGGTCATACGCGGACGCCGAGGCCCAGTCCGTG
$PecA_{A89D}$-A88E	CCGGGTCATACGAGGACGCCGAGGC
PecA-A84D	CTGAACTCATCGGACGGGTCATACGCG
MMAR_2933 LF	TTTTTTTTCACAAAGTGGCCGACACTCTTGATGTTGA
MMAR_2933 LR	TTTTTTTTCACTTCGTGGCTGATCGCCTGGTATTCCT
MMAR_2933 RF	TTTTTTTTCACAGAGTGACTACCAGCCGACGGTCATT
MMAR_2933 RR	TTTTTTTTCACCTTGTGCGTGGCTTCACTGCCTACTT
